# Veterinary nurses report good interprofessional collaboration with veterinarians, as measured by the Nurse–Physician Collaboration Scale

**DOI:** 10.1002/vetr.70549

**Published:** 2026-03-29

**Authors:** Samantha J. Fontaine, Kelly Cadman, Euan D. Bennet, Dorothy E. F. McKeegan

**Affiliations:** ^1^ University of Glasgow Glasgow UK; ^2^ University of Edinburgh Roslin UK

## Abstract

**Background:**

Veterinary practices provide exceptional patient care and client support when there is effective teamwork between veterinary nurses and veterinarians. However, the frequency of this interprofessional collaboration has not been measured.

**Methods:**

A cross‐sectional online survey, using the Nurse‒Physician Collaboration Scale to measure the frequency of the collaborative behaviours of joint decision making (JDM), cooperativeness (C) and sharing patient information (SPI), was administered to registered and student veterinary nurses. A multivariable logistic regression model was constructed for each behaviour construct.

**Results:**

Veterinary nurses (*n* = 835) self‐reported collaboration with veterinarians at item level as either ‘good’ or ‘mixed’ (defined as a median item score ≤2 or >2‒3.5). Overall, the median (interquartile range) scores by collaborative behaviour construct were 2 (2‒3) for JDM, 2 (1‒3) for C and 2 (2‒3) for SPI. Multivariable analysis found that role and practice type were significantly associated with participants reporting good experiences in each behaviour construct.

**Limitations:**

The survey did not capture the perspectives of veterinarians for comparison.

**Conclusion:**

Collaboration between veterinary nurses and veterinarians is good, but improvements in all behaviour constructs could be made. Efforts should focus on improving communication and increasing the opportunities to mutually participate in patient care decision making.

## INTRODUCTION

Interprofessional collaboration between veterinarians and veterinary nurses is fundamental to the successful running of veterinary practices, the provision of safe holistic patient care and exceptional client support.^1^, ^2^ Patient care and safety is achieved through interdisciplinary, not individual, care, and all health professionals require excellent intercommunication to prevent adverse events from occurring.^3^

Collaboration occurs when two or more individuals work together to achieve a desired outcome,^4^ which for veterinary teams is a shared directive to deliver high standards of care to improve the health and welfare of animals.^5^ However, for true collaboration to occur, there must be shared responsibility and decision making, accountability and communication.^1^ Considered key to collaboration is a non‐hierarchical complementary relationship where there is an equal share of power.^6^, ^7^ Hierarchical organisational structures are common within the veterinary profession and although this has been described as a barrier to interprofessional working, it has also been shown to enable improved communication by providing a structured pathway from the top down.^8^, ^9^

In the modern multidisciplinary veterinary team, collaboration and teamwork are important professional qualities described in the Code of Professional Conduct for both veterinarians and registered veterinary nurses (RVNs).^10^, ^11^ The Royal College of Veterinary Surgeons (RCVS) and the British Veterinary Association have defined that veterinary teams should be directed by the veterinarian who represents the ‘hub’ of a ‘vet‐led team’.^12^, ^13^ Implementing a stable and functional team approach within the practice environment allows everyone's strengths to be utilised, aids better understanding of roles and role differentiation, improves job satisfaction and creates an efficient workplace with a commitment to patient care and the client experience.^5^, ^14^ Veterinary teamwork and interprofessional collaboration can be enabled through effective communication, shared goals and values, trust, mutual support and embracing diversity and different perspectives; personal attributes considered important to both veterinary professionals and their clients.^8^, ^14^ However, differences in professional motivations can create tension between the professions, with veterinarians typically being more focused on cure, and veterinary nurses more focused on care.^8^ The spatial and temporal nature of veterinary work, which can result in colleagues working in different areas of a clinic or across multiple branches of an organisation, may also challenge collaboration.^8^ Theoretically, teamwork should be easier to instil in veterinary practices when compared to healthcare services, as there are fewer professionals working together, with less services needing to be provided to a smaller number of patients.^15^ However, the proposed expansion of the vet‐led team to include other allied professionals, such as veterinary behaviourists and physiotherapists, may impact on this.^12^, ^13^

It is important to understand the perceptions, barriers and enablers of interprofessional relationships, and several instruments have been reported to measure collaborative behaviours of nurses and physicians. Examples include the Collaborative Practice Scale,^16^ ICU Nurse‒Physician Questionnaire,^17^ Satisfaction About Care Decisions,^18^ Nurses’ Opinion Questionnaire^19^ and the Jefferson Scale of Attitudes towards Physician‒Nurse Collaboration.^20^ The Nurse‒Physician Collaboration Scale (NPCS) developed by Ushiro^21^ is particularly valuable since it measures the frequency of collaborative behaviours of both nurses and physicians in the process of patient‐centred care rather than just attitudes towards collaboration. It comprises three constructs of collaborative behaviour: cooperativeness (C), defined as mutual support and open communication; joint participation in the cure/care decision‐making process (joint decision making [JDM]); and sharing patient information (SPI). It has been used to measure the frequency of collaborative behaviours in acute care settings and dental hospitals in Japan, China and North America.^21^, ^22^, ^23^

Most of the literature on nurse‒physician collaboration is presented from a nursing perspective, and positive relationships have been identified between nurses’ attitudes towards collaboration and their professional values, level of education and hospital department.^24^, ^25^, ^26^ A review of the wider nurse‒physician collaboration literature reveals mixed findings about which of the two professions typically behave more collaboratively or perceive collaboration to be good.^27^ A meta‐analysis of 51 studies showed that while physicians perceive there to be more collaboration than nurses, nurses have a more positive attitude towards collaboration than physicians.^28^ The contrasting findings across studies may reflect different definitions of collaboration and methodological differences in the instruments; emphasising the need to understand if they are assessing attitude to, perception of or frequency of collaboration.^28^ To draw meaningful conclusions about individual professionals’ experiences of collaboration, standardisation is required.^27^

Despite the reported benefits of effective collaboration between nurses and physicians in healthcare settings, including improved patient outcomes, decreased mortality, better job satisfaction, improved retention and decreased economic impacts,^23^, ^26^ there has been little research of this phenomenon conducted in the veterinary profession. Obvious comparisons can be drawn between the nurse‒physician relationship and the veterinary nurse‒veterinarian relationship. However, assumptions that veterinary nurse perceptions of, or participation in, collaboration are the same as registered nurses should be made with caution, due to differences in patient advocacy, role responsibilities and degree of autonomy, with veterinary nursing only having achieved professional status in 2015.^29^

The aim of this research was to assess the frequency of veterinary nurse‒veterinarian collaboration from the perspective of veterinary nurses (including student veterinary nurses [SVNs]), and to determine which personal and professional factors impact on collaboration behaviours. Specifically, our objectives were to determine whether the veterinary‐adapted NPCS^21^ instrument is reliable and valid when administered to veterinary nurses and measure the reported frequency of the three NPCS behavioural constructs of collaboration: JDM, C and SPI. We also wanted to investigate the effect of demographic and clinical practice variables on veterinary nurse collaboration scores across the three constructs.

It is anticipated that these baseline values will be used in future studies to compare veterinarian collaboration scores at a macro level, or veterinary nurse scores at a meso level within individual organisations.

## METHODS

This cross‐sectional survey study recruited RVNs and SVNs, hereto collectively referred to as veterinary nurses, currently working in clinical practice. Sample size was calculated from an estimated population of 21,360 (comprising 18,480 RVNs in 2020 and 2880 new registrants between 2020 and 2021 as an estimate of the student population).^30^ For a confidence level of 95% and a margin of error of 5%, the required sample size was 377. A convenience non‐probability sample was used, and the survey was distributed by email to veterinary nurses who had opted into the RCVS research database, with further snowball sampling via professional organisations, veterinary publications and social media. The survey was open for 6 weeks in February 2020 and was conducted with approval from the University of Glasgow College of Medical, Veterinary and Life Sciences Ethics Committee. Consent was indicated by the completion of the survey and the responses were anonymous.

### Instrument

The survey instrument was administered using Jisc Online Surveys^31^ and comprised three sections: (1) demographic section collecting data on age, gender, level of veterinary nursing qualification, job role and type of practice where currently employed; (2) the veterinary‐adapted NPCS^21^; and (3) a free‐text question asking participants if they felt overall that their opinions and knowledge are listened to by veterinary surgeons and are acted on to influence patient care (the results of which are outside the aims of this paper and will be reported separately). Participants had the option of opting out of specific questions if desired. Permission to use and modify the NPCS was obtained from the author. As the NPCS was designed to assess the collaborative behaviours between nurses and physicians, some adaptations were required to change terminology from ‘physician’ to ‘veterinarian’ and ‘patient’ to ‘client’, and three questions were removed that were deemed by the researchers as not appropriate for the role of veterinary nurses in the care of animal patients.

Similar job roles and similar practice types were grouped together for the purpose of analysis: veterinary nurses and locums were combined as ‘veterinary nurse’, senior nurses and head nurses, as ‘senior nurse’, specialised and co‐ordinator roles, as ‘specialised nurse’, and practice managers, partners and clinical directors, as ‘senior manager’. For practice type categories, general practices and charities were grouped together as ‘general practice’, and referral hospitals and hospitals that were described as mixed general and referral were grouped as ‘referral hospital’. For species, large animal and mixed animal were grouped as ‘mixed’.

The NPCS comprises three collaborative behaviour constructs: JDM, C and SPI. The 24 individual items that were part of these constructs are described in Table 1. Participants scored each item using a five‐point Likert scale (1 = always, 2 = usually, 3 = sometimes, 4 = rarely, 5 = never), thus lower scores equate to a greater frequency of reported collaborative behaviour. Due to the ordinal nature of the Likert scale data, median scores were calculated for each construct (JDM, C and SPI). Item median scores for perceived frequency of collaboration were categorised as ‘good’ when 2 or less, mixed when more than 2.0 to 3.5, and poor when more than 3.5. The original NPCS content validity and internal consistency reliability was satisfactory, with a Cronbach's alpha coefficient above 0.80 and test‒retest coefficients above 0.7 for all the constructs.^21^ Piloting was conducted with four RVNs to test face validity, ensure readability and calculate the mean time for survey completion.

**TABLE 1:** Demographic data of all the participants.

Demographic variable	*N* = 835	
	*n*	%
**Age (years)**		
18‒30	405	48.5
31‒45	326	39.0
45+	103	12.3
Prefer not to say	1	0.1
**Gender**		
Female	801	95.9
Male	28	3.4
Non‐binary	4	0.5
Prefer not to say	2	0.2
**Highest level of veterinary nursing education**		
Diploma (or equivalent)	588	70.4
Bachelor's degree	212	25.4
Postgraduate degree	8	1.0
Prefer not to say	27	3.2
**Highest level of non‐veterinary nursing education**		
None	333	39.9
Diploma (or equivalent)	335	40.1
Bachelor's degree	125	15.0
Postgraduate degree	19	2.3
Prefer not to say	23	2.8
**Time qualified (years)**		
<1	33	4.0
1‒6	345	41.3
7‒14	218	26.1
15+	239	28.6
**Employment status**		
Full‐time	636	76.2
Part‐time	191	22.9
Prefer not to say	8	1.0
**Role**		
Student veterinary nurse	64	7.7
Veterinary nurse	476	57.0
Locum	3	0.4
Senior/head nurse	220	26.3
Specialised nurse role	56	6.7
Co‐ordinator	2	0.2
Practice manager	12	1.2
Clinical director	1	0.1
Practice partner	3	0.4
**Practice type**		
General practice	515	61.7
General hospital	141	16.9
Referral hospital	89	10.7
University hospital	39	4.7
Emergency clinic	40	4.8
Charity practice	8	1.0
Mixed general practice/referral hospital	3	0.4
**Patient species**		
Small animal	721	86.3
Equine	20	2.4
Large animal	3	0.4
Exotics	3	0.4
Mixed	88	10.5

### Data analysis

Validity of the veterinary‐adapted instrument was tested using Cronbach's alpha coefficient, which is a measure of internal consistency. Acceptable values of alpha have been reported to range from 0.70 to 0.95, although a maximum alpha value of 0.90 has been suggested.^32^

All the responses were included in the final analysis, and where data were missing for the variables, the sample size is reported. Data were cleaned and ‘other’ responses were recoded into existing or new categories. NPCS data from each item were grouped into the behaviour constructs: JDM (11 questions), C (six questions) and SPI (seven questions).

Comparisons between overall construct scores were made using the Kruskal‒Wallis test. Three multivariable logistic regression models were constructed: one for each behaviour construct. The outcome of each model was the binary variable ‘good experience’, cases were those participants whose mean score for all questions in the particular behaviour construct was 2 or less, controls were those participants whose mean score was greater than 2. Models were constructed using a manual stepwise backward‐removing process, starting with all demographic variables as candidate input variables. Potential for confounding was investigated between factors retained in the final models, as well as between those retained and those rejected during the model‐building process. Significance was set at a *p*‐value of less than 0.05. Data were analysed using Minitab 21 and R 4.4.0.^33^, ^34^

## RESULTS

The total number of responses was 850. Of these, 15 were excluded as respondents were not currently working in clinical practice, leaving a sample size of 835.

The sample comprised 92.3% (*n* = 771) RVN and 7.7% (*n* = 64) SVN. The majority identified as female (*n* = 801, 95.9%), and the most common age groups were 18‒30 years (*n* = 405, 48.5%) and 31‒45 years (*n* = 326, 39.0%). A diploma level qualification, or equivalent, was held by most (*n* = 588, 70.4%) respondents, but a quarter (*n* = 212, 25.4%) held a veterinary nursing degree. For those also holding qualifications in a non‐veterinary nursing subject, 15% (*n* = 125) held a bachelor's degree and 2.3% (*n* = 19) held a master's level qualification or higher. Overall, 39.4% (*n* = 329) of respondents were educated to degree level. Just under half (*n* = 345, 41.3%) had been an RVN for 1‒6 years, with 26.1% (*n* = 218) having been an RVN for 7‒14 years, and 28.6% (*n* = 239) having been an RVN for 15 years or more. The majority (*n* = 636, 76.2%) worked full‐time, held the job role of veterinary nurse (*n* = 476, 57.0%) and worked with small animal patients (*n* = 721, 86.3%) in a general practice setting (*n* = 515, 61.7%). Full demographic descriptive statistics are presented in Table 1.

### Instrument validity and reliability

The adapted NPCS retained excellent internal consistency of the scale, with Cronbach's alpha coefficients of 0.91 for JDM, 0.84 for C and 0.87 for SPI. When Cronbach's alpha coefficients of the item‐total correlations were compared with those obtained when an item had been eliminated, no items were found to improve the coefficient values. The item‐adjusted total correlations ranged from 0.54 to 0.76 for JDM, from 0.49 to 0.69 for C and from 0.57 to 0.68 for SPI. Item‐total correlations indicated good discrimination and supported that items on the same factor were measuring the same construct.

### Collaboration behaviour constructs

NPCS item scores and overall behaviour construct scores are presented as median and interquartile range (IQR), as shown in Table 2. The median (IQR) values by behaviour construct were 2 (2‒3) for both JDM and SPI, and 2 (1‒3) for C. Kruskal‒Wallis comparison of the median scores of all constructs showed they were significantly different from each other (*p* < 0.001), and from the IQR, C appeared to be the most positively scored construct.

**TABLE 2:** Veterinary nurse (*n* = 835) median item scores for the veterinary‐adapted Nurse‒Physician Collaboration Scale (NPCS) collaboration behaviour constructs of joint decision making (JDM), cooperativeness (C) and sharing patient information (SPI).

NPCS collaboration behaviour construct and items	Survey question number	Median score (IQR)
**JDM**		
(JDM1) The nurses and veterinarians exchange opinions to resolve problems related to patient care.	10	2 (2‒3)
(JDM2) In the event of a disagreement about the future direction of a patient's care, the nurses and veterinarians hold discussions to resolve differences of opinion.	11	2 (2‒4)^a^
(JDM3) The nurses and veterinarians discuss whether to continue a certain treatment when that treatment is not having the expected effect.	13	2 (2‒3)
(JDM4) When a patient is to be discharged from the hospital, the nurses and veterinarians discuss the lifestyle regime the patient needs to follow.	14	2 (2‒3)
(JDM5) When confronted by a difficult patient, the nurses and veterinarians discuss how to handle the patient.	15	2 (1‒3)^b^
(JDM6) The nurses and veterinarians discuss the problems a patient has.	17	2 (1‒3)^b^
(JDM7) The nurses and veterinarians together consider their proposals about the future direction of patient care.	18	2 (2‒3)
(JDM8) In the event a patient develops unexpected side effects or complications, the nurses and veterinarians discuss countermeasures.	19	2 (1‒3)^b^
(JDM9) The future direction of a patient's care is based on a mutual exchange of opinions between the nurses and veterinarians.	21	2 (2‒3)
(JDM10) The nurses and veterinarians seek agreement on signs that a patient can be discharged.	22	2 (2‒3)
(JDM11) The nurses and veterinarians discuss how to prevent medical care accidents.	24	2 (2‒3)
**C**		
(C1) The nurses and veterinarians greet each other every day.	12	1 (1‒2)^b^
(C2) The nurses and veterinarians take into account each other's schedule when making plans to treat a patient together.	16	3 (2‒4)^a^
(C3) The nurses and veterinarians can easily talk about topics other than those related to work.	20	2 (1‒2)
(C4) The nurses and veterinarians can freely exchange information or opinions about matters related to work.	23	2 (1‒3)
(C5) The nurses and veterinarians help each other.	29	2 (1‒3)
(C6) The nurses and veterinarians show concern for each other when they are very tired.	33	2 (1‒3)
**SPI**		
(SPI1) The nurses and veterinarians all know what has been explained to a client about their pet's condition or treatment.	25	3 (2‒3)^a^
(SPI2) The nurses and veterinarians share information to verify the effects of treatment.	26	2 (2‒3)
(SPI3) The nurses and veterinarians have the same understanding of the future direction of the patient's care.	27	2 (2‒3)
(SPI4) In the event of a change in treatment plan, the nurses and veterinarians have a mutual understanding of the reasons for the change.	28	2 (2‒3)
(SPI5) The nurses and veterinarians check with each other concerning whether a patient has any signs of side effects or complications.	30	2 (1‒2)^b^
(SPI6) The nurses and veterinarians share information about a client's reaction to explanations of their pet's disease status and treatment methods.	31	2 (2‒3)
(SPI7) The nurses, veterinarians and clients have the same understanding of the patient's ability for cure and care.	32	2 (2‒3)

*Note*: Lower values indicate greater perceived frequency of collaboration. Mean scores 2.0 or less were categorised as good, more than 2.0 to 3.5 as mixed, and more than 3.5 as poor collaboration.

Abbreviation: IQR, interquartile range.

^a^ Item(s) with the least frequently reported collaborative behaviour for each construct.

^b^ Item(s) with the most frequently reported collaborative behaviour for each construct.

Only two items (C2 and SPI1) had median scores categorised as ‘mixed’ collaboration (mean score >2‒3.5), with the remaining 22 items categorised as ‘good’ collaboration (mean score ≤2). No items were categorised as ‘poor’ collaboration (mean score >3.5). The lowest scoring item, reporting the greatest frequency of collaboration, was C1: ‘the nurses and veterinarians greet each other every day’ (median = 1, IQR = 1‒2), and the least frequently scoring item was C2: ‘the nurses and veterinarians take into account each other's schedules when making plans to treat a patient together’ (median = 3, IQR = 2‒4).

At the demographic variable level, the median (IQR) scores for the three behaviour constructs were good (Table S1), except for three factors within the JDM construct that were mixed: those whose highest non‐veterinary nursing qualification was an MSc or PhD (median = 3, IQR 2‒3), those who worked in a university hospital (median = 3, IQR = 2‒3), and those whose main species cared for was equids (median = 3, IQR = 2‒4).

In each of the three logistic regression models, two demographic factors were found to be statistically significantly associated (*p* < 0.05) with the outcome ‘good experience’: role and practice type (Figure 1). The full results of the models are shown in Table 3.

**FIGURE 1:**
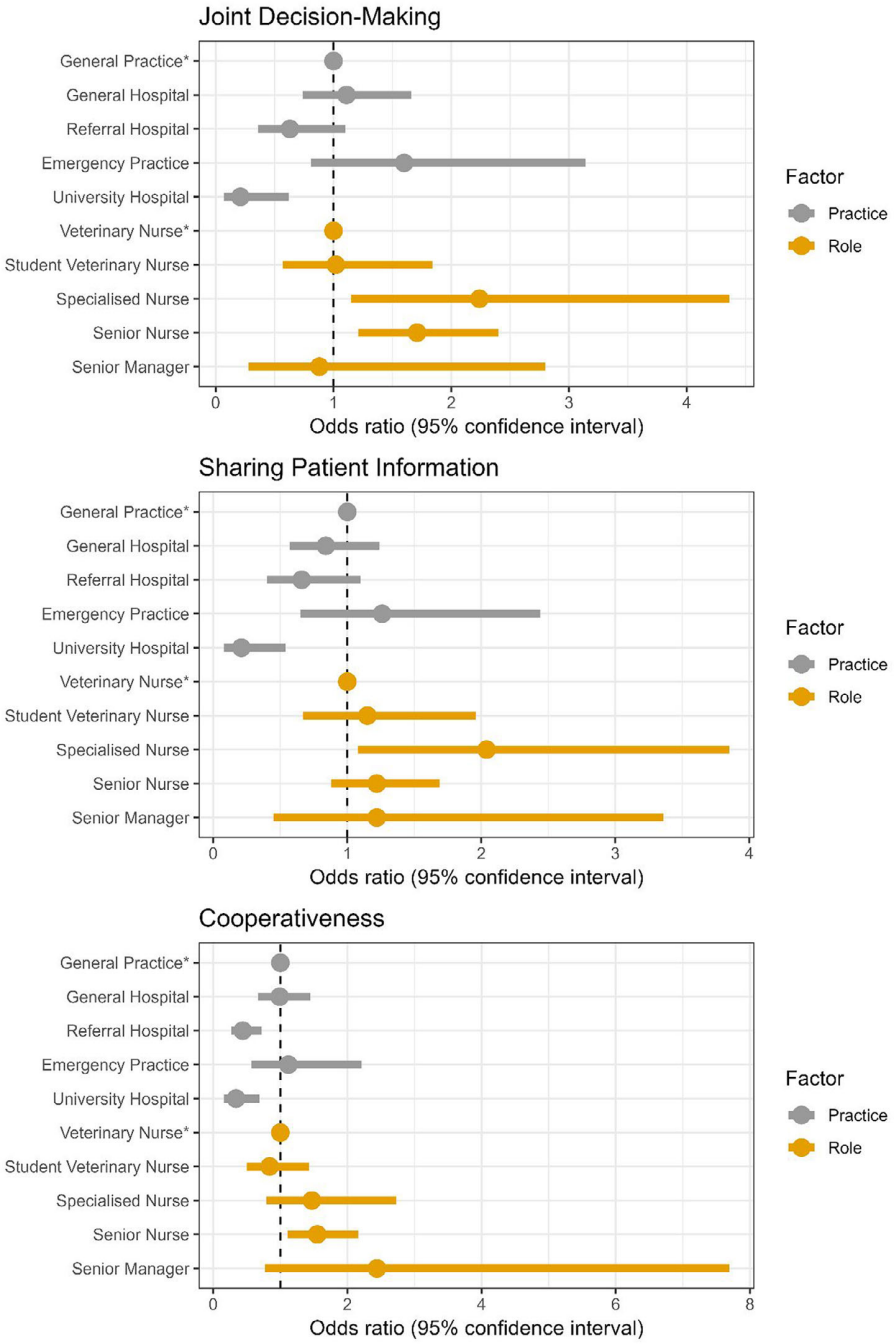
Three dot‐and‐whisker plots—one per behavioural construct—showing the results of the three multivariable linear regression models. The points show the point estimates of the odds ratios with which each factor level is associated with each respective outcome. The error bars show the 95% confidence intervals. The vertical dashed line is located at an odds ratio of 1 and corresponds to the reference category for each factor.

**TABLE 3:** Results of three multivariable logistic regression models for the outcome constructs ‘joint decision making (JDM)’, ‘sharing patient information (SPI)’ and ‘cooperativeness (C)’, assessed as a binary outcome of ‘good experience reported by participant’.

Demographic factor	Participants	Mean score 2 or less (%)	Odds ratio	95% confidence interval	*p*‐Value
**Outcome: mean score 2 or less for JDM questions**					
Role					
Veterinary nurse*	479	127 (26.5%)	1	‒	‒
Senior manager	16	4 (25%)	0.88	0.28‒2.8	0.8
Senior nurse	220	84 (38.2%)	1.71	1.21‒2.4	0.002
Specialised nurse	56	20 (35.7%)	2.24	1.15‒4.36	0.018
Student veterinary nurse	64	18 (28.1%)	1.02	0.57‒1.84	0.9
Practice type					
General practice*	526	162 (30.8%)	1	‒	‒
Emergency practice	39	17 (43.6%)	1.6	0.81‒3.14	0.2
General hospital	141	47 (33.3%)	1.11	0.74‒1.66	0.6
Referral hospital	90	23 (25.6%)	0.63	0.36‒1.1	0.1
University hospital	39	4 (10.3%)	0.21	0.07‒0.62	0.005
**Outcome: mean score 2 or less for SPI questions**					
Role					
Veterinary nurse*	479	176 (36.7%)	1	‒	‒
Senior manager	16	7 (43.8%)	1.22	0.45‒3.36	0.7
Senior nurse	220	92 (41.8%)	1.22	0.88‒1.69	0.2
Specialised nurse	56	25 (44.6%)	2.04	1.08‒3.85	0.03
Student veterinary nurse	64	26 (40.6%)	1.15	0.67‒1.96	0.6
Practice type					
General practice*	526	216 (41.1%)	1	‒	‒
Emergency practice	39	19 (48.7%)	1.26	0.65‒2.44	0.5
General hospital	141	53 (37.6%)	0.84	0.57‒1.24	0.4
Referral hospital	90	32 (35.6%)	0.66	0.4‒1.1	0.1
University hospital	39	6 (15.4%)	0.21	0.08‒0.54	0.001
**Outcome: mean score 2 or less for C questions**					
Role					
Veterinary nurse*	479	251 (52.4%)	1	‒	‒
Senior manager	16	12 (75%)	2.44	0.77‒7.69	0.1
Senior nurse	220	139 (63.2%)	1.55	1.11‒2.16	0.009
Specialised nurse	56	28 (50%)	1.47	0.79‒2.73	0.2
Student veterinary nurse	64	32 (50%)	0.84	0.5‒1.43	0.5
Practice type					
General practice*	526	308 (58.6%)	1	‒	‒
Emergency practice	39	24 (61.5%)	1.12	0.57‒2.21	0.7
General hospital	141	81 (57.4%)	0.99	0.67‒1.45	0.9
Referral hospital	90	36 (40%)	0.44	0.27‒0.72	0.001
University hospital	39	13 (33.3%)	0.34	0.16‒0.69	0.003

*Note*: For each categorical variable, asterisk (*) indicates the reference category.

Compared to veterinary nurses, senior nurses were at increased odds of reporting a good collaborative experience (odds ratio [OR] = 1.71, 95% confidence interval [CI] 1.21–2.40 for JDM; OR = 1.55, 95% CI 1.11–2.16 for C). Specialised nurses were also at increased odds of reporting a good collaborative experience compared to veterinary nurses (OR = 2.24, 95% CI 1.15–4.36 for JDM; OR = 2.04, 95% CI 1.08–3.85 for SPI).

Compared to general practice, working at a university hospital was associated with reduced odds of participants reporting a good collaborative experience for all three constructs, with ORs of 0.21 (95% CI 0.07–0.62) for JDM, 0.21 (95% CI 0.08–0.54) for SPI and 0.34 (95% CI 0.16–0.69) for C. Working at a referral hospital was also associated with reduced odds of participants reporting a good experience for C outcomes (OR = 0.44, 95% CI 0.27–0.72).

No confounding was detected between any variables retained or rejected during the model‐building process. While investigating potential confounding, the intersection between role and practice type was also investigated. Table 4 shows the number and proportion of each participant role by practice type and shows that broadly similar proportions of three of the five roles were reported to work at each practice type. The role of senior manager was found only in general practice and general hospital practice types, and all but one of these 16 participants worked in a general practice and charity setting. The role of specialised nurse was found in all five practice types but with a different distribution compared to most other roles. Neither the senior manager nor the specialised nurse role was found to be statistically significantly different to the reference veterinary nurse role in any of the models.

**TABLE 4:** Distribution of participant role by practice type.

Practice type	Veterinary nurse	Senior manager	Senior nurse	Specialised nurse	Student veterinary nurse
General practice	314 (59.7%)	15 (2.9%)	152 (28.9%)	5 (1.0%)	40 (7.6%)
Emergency practice	22 (56.4)	0 (0%)	9 (23.1%)	6 (15.4%)	2 (5.1%)
General hospital	80 (56.7%)	1 (0.7%)	33 (23.4%)	8 (5.7%)	19 (13.5%)
Referral hospital	43 (47.8%)	0 (0%)	20 (22.2%)	25 (27.8%)	2 (2.2%)
University hospital	20 (51.3%)	0 (0%)	6 (15.4%)	12 (30.8%)	1 (2.6%)

## DISCUSSION

Collaboration is a vital component of effective interprofessional relationships, and the veterinary‐adapted NPCS reliably measures the frequency of collaborative behaviours between veterinary nurses and veterinarians. This study has shown that veterinary nurses report a good frequency of collaboration with veterinarians at a behavioural construct level, with mostly good collaboration across individual items within the constructs. Although it is promising to see that no items or constructs were categorised with a poor frequency of collaboration, from a veterinary nurse's perspective, improvements in item and construct scores are still possible.

The behavioural construct of C was scored most positively by participants when examining the IQRs. This finding should be considered positive given that the items within it relate to mutual civility and support. Incivility is reported to occur within the veterinary profession, with veterinary nurses experiencing significantly more incivility from co‐workers and senior colleagues compared to veterinarians.^35^ Despite this, the veterinary nurses in this study appear to perceive professional relationships with veterinarians as mostly collegial.

Direct comparisons to other studies are somewhat challenging due to them reporting mean construct scores and this study reporting median scores, but it appears that veterinary nurses differ somewhat from registered nurses, for whom SPI was the most frequently occurring collaborative behaviour reported in studies undertaken in an acute care hospital in the United States^22^ and in a dental hospital in China.^23^ Nair et al.^22^ found physicians reported similar findings to the veterinary nurses in this study, scoring the construct of C most positively. The authors proposed that this was due to the level of familiarity the physicians had with the nurses, given the length of time qualified and practicing in the same hospital (most were qualified for more than 10 years and had been at the hospital for more than 5 years). Although just over half of the veterinary nurses in this sample had been qualified for more than 7 years, it is impossible to draw similar conclusions about the familiarity of colleagues as it is not known how long they had spent in their current practice. This may be a variable of interest in future studies.

It is posited that SPI is reported most frequently by nurses due to the need for them to share pertinent patient details with physicians and to advocate effectively for the patient^22^; a duty that, arguably, veterinary nurses similarly hold. This would suggest that there may be unique obstacles or inefficiencies for veterinary nurses that are creating barriers to patient information being transferred as freely or as easily as it could. When scrutinising each item in this construct, the item that scored lowest pertained to knowledge of what was explained to clients (SPI1). As the veterinarian typically interacts independently with the client to make treatment plans, these findings suggest that veterinary nurses are not always made privy to this information afterwards. In contrast to registered nurses, the patient cannot update the veterinary nurse of what was discussed either. This creates a risk that veterinarians and veterinary nurses have divergent mental models of the goals of care, which may result in interpersonal conflict or poorer patient outcomes.^36^

A bidirectional flow of information is essential for teams to effectively solve problems and make informed clinical decisions; therefore, veterinarians should endeavour to communicate all aspects of the patient's treatment plan with veterinary nurses. Communication problems within teams can negatively affect patient outcomes, causing errors in the delivery of care that can result in treatment delay, harm or death.^37^, ^38^ Poor communication is a commonly reported cause of veterinary medical errors, with incidence rates ranging from 5% to 30%.^37^, ^39^, ^40^ For veterinary staff more generally, this poor communication can cause adverse non‐clinical outcomes such as inefficiency and frustration.^25^, ^41^ Creating a positive practice culture that supports interprofessional relationships and open channels of communication should therefore be a priority for practices and managers.^38^, ^42^, ^43^ Improving the frequency and ease of SPI through supported and structured communication opportunities that allow a bidirectional flow of patient information, such as handovers and ward rounds, may be of benefit.^44^, ^45^, ^46^

In this study, JDM collaborative behaviour did not have the poorest reported frequency, as reported by registered nurses. It has been postulated that the poorer score for this construct in both registered nurses and physicians is due to negative professional opinions held by physicians regarding the perceived inferior knowledge and skills of nurses, and that deferential behaviours used by nurses made physicians more likely to dismiss their ideas and exclude them from decision making.^22^ The results from the present study suggest that the veterinary nurse‒veterinarian relationship may be based on better levels of mutual professional respect. The three best scoring items in the this construct were JDM5 (discuss how to handle difficult patients), JDM6 (discuss patient problems) and JDM8 (discuss unexpected side effects and complications). Veterinary nurses often lead the animal handling and restraint, and it is unsurprising that they perceive that collaboration occurs frequently for this activity, especially when the safety of staff and patients is at risk. Another core competency of veterinary nurses is to monitor and assess patients, and it is positive that there also appears to be good collaboration when discussing patient problems and when discussing actions when unexpected changes to patients’ conditions arise. The poorest scoring item (JDM2) was related to discussing disagreements in the future direction of patient care, indicating that veterinary nurses feel less influential in this activity. Although less obvious in this study, nurse exclusion from case discussions and treatment decisions has previously been described.^1^, ^42^

Joint decision making requires interprofessional relationships based on trust and mutual respect, where the opinions of both professional groups are considered and acted upon.^47^ Hierarchy theory proposes that those in higher positions of power are less likely to express a need for collaboration, with nurses tending to view collaboration as more important than physicians.^48^, ^49^ Despite this hierarchy, there is an expectation that veterinarians hold core competences around communicating effectively with colleagues, can work effectively as a member of an interprofessional team and understand what each profession contributes. However, these competencies may not fully align to the training methods used to educate veterinarians, which traditionally follow a similar model to that of medical professional education, where physicians are trained to have independent decision‐making skills. By contrast, nurses are trained to make decisions interdependently with physicians.^50^ The interprofessional education (IPE) approach to veterinary and veterinary nurse education may support the development of respect, teamwork and collaborative behaviours at early stages of training, which may in turn help to break down the traditional hierarchy that exists between the two professions.^51^ It has already gained traction in recent years, although it is still unclear whether the positive changes in professional attitudes developed during this training persist long term after graduation.^51^, ^52^ Continuing professional development for veterinarians on the role of veterinary nurses and the value of interprofessional teams may also be beneficial in improving interprofessional relationships, and RCVS initiatives to raise awareness of delegation from veterinarians to veterinary nurses should be implemented.^53^

In the multivariable models, role was one of two major factors associated with participants reporting a good experience. Those in senior and specialised nurse roles, and senior management roles, were more likely to report a good experience across the three outcomes compared to those in veterinary nurse roles, although this difference was not always significant. Given that senior and specialised nurses and senior nurse managers typically work closely with senior veterinarians to deliver patient care and to influence practice policy and procedure, this is an unsurprising finding and may be a factor in why head nurses are less likely to resign.^54^

The second major factor associated with participants reporting a good experience was practice type, with similar trends seen across all three collaborative behaviours and those in emergency and general practice scoring better than those in referral and university hospitals. In the multivariable models, participants at university hospitals reported significantly worse experiences for all three outcomes compared to participants in general practice. These results suggest that collaboration within larger hospital environments is perceived to be more challenging for veterinary nurses. Effective interprofessional relationships in such environments may be hindered by many factors, including larger size of the organisation and its teams, cross‐team involvement in an individual patient's care, staff rotating through departments, staff siloed within departments that may be geographically remote to each other, greater specialisation, diverging agendas for veterinarians and veterinary nurses, and a greater hierarchy between specialist veterinarians and veterinary nurses.^8^, ^55^ The smaller team sizes within general and emergency practice, and the high‐intensity nature of emergency veterinary medicine, may support more effective teamwork and cooperation to achieve desired outcomes. The effect of different hospital departments on experiences of nurse‒physician collaboration has been shown by several authors,^25^, ^27^, ^28^ with hospital intensive care units evidencing greater frequency and more successful collaboration due to the necessity for excellent communication, teamwork and for nursing staff to work independently due to the nature of the patient care required.^56^, ^57^

The main limitation of this study is that only the perspective of veterinary nurses was obtained, and no conclusions can be drawn about how these relate to the perspectives of veterinarians. In addition, although the NPCS was not designed for use by veterinary professionals, we assumed similarity in the roles and relationship of nurses and physicians. Despite requirements to adapt the instrument slightly, it retained good internal consistency, and the instrument was valid and reliable when used with veterinary nurses. Future work on the perspective of veterinarians would be of merit to determine if the frequency of collaborative behaviours is similar or different to veterinary nurses and could help provide further insight into these interprofessional relationships. A gender bias was present in the sample, although this mirrors the strong gender bias within the veterinary professions, especially within veterinary nursing.^58^ Response bias could have occurred due to the self‐reporting nature of the instrument, and there was a risk that veterinary nurses with strong negative interprofessional experiences would be more likely to participate in the study. An attempt was made to limit this effect by asking participants to answer in relation to a general view of their professional experiences, and not to focus on specific incidents. The fact that the perceptions of the frequency of collaboration were positively skewed would suggest that participants followed this instruction, and that negative response bias did not occur.

The results of this preliminary study reveal that veterinary nurses perceive the frequency of collaboration with veterinary surgeons as good, but there is room for improvement across all three behaviour constructs. Perceptions of collaboration may be affected by veterinary nurse's role and practice type, and so acknowledgement of these contextual differences is necessary to foster more effective interprofessional collaboration for some individuals. Efforts could focus on improving communication and increasing the opportunities for veterinary nurses and veterinarians to share information regarding patients’ care and, wherever possible, mutually participate in patient care decision making. This study has implications for veterinary practice and the successful collaboration of veterinary professionals, and it is essential that leaders commit to achieving effective working relationships to support a positive practice culture and good patient outcomes. Effective communication strategies should be embedded within practices and collaborative behaviours routinised. Veterinary educators should also encourage and facilitate opportunities for IPE where veterinary and veterinary nurse students can develop respectful and effective professional relationships that are focused on effective collaboration and that flatten the traditional hierarchy. The veterinary‐adapted NPCS is a potentially useful instrument to measure changes in interprofessional collaboration over time, and future work could investigate the impact of IPE and the implementation of interventions to improve collaboration. Greater evidence of the value of collaboration between veterinary nurses and veterinarians is required, and future variables of interest could include patient care outcomes, career satisfaction and employee retention.

## AUTHOR CONTRIBUTIONS

The second author, Kelly Cadman, conducted this study in part fulfilment of the MSc Advanced Practice in Veterinary Nursing at the University of Glasgow. Samantha J. Fontaine and Dorothy E.F. McKeegan were primary and secondary supervisors and contributed to the study design, study execution and data analysis and interpretation. Euan D. Bennet provided statistical support and performed additional statistical analysis. All the authors contributed to the preparation of this manuscript and gave final approval to the manuscript.

## CONFLICT OF INTEREST STATEMENT

The authors declare they have no conflicts of interest.

## ETHICS STATEMENT

Ethical approval for the study involving human participants was granted by the University of Glasgow, College of Medical, Veterinary and Life Sciences Research Ethics Committee (application number 200190098).

## Supporting information

Supporting Information

## Data Availability

The data that support the findings of this study are available from the corresponding author, S.J.F., upon reasonable request.
