# Supplementary material for: Veterinary nurses report good interprofessional collaboration with veterinarians, as measured by the Nurse–Physician Collaboration Scale
Source: Vet Rec. 2026 Mar 29;199(10):e471–81. doi: 10.1002/vetr.70549 (PMC13768724; doi:10.1002/vetr.70549)
Supplement: Supplementary file 1 [file VETR-199--s001.docx]

Supplementary Table S1. Median scores of collaborative behaviour construct scores by participant demographic variables.

|  |  | ***Joint Decision Making*** | ***Cooperativeness*** | ***Sharing Patient Information*** |
| --- | --- | --- | --- | --- |
| **Variable** | **n** | **Median score (IQR)** | **Median score (IQR)** | **Median score (IQR)** |
| ***Age (years):*** | ***834*** |  |  |  |
| 18-30 years | 405 | 2 (2 - 3) | 2 (1 - 3) | 2 (2 - 3) |
| 31-45 years | 326 | 2 (2 - 3) | 2 (1 - 3) | 2 (2 - 3) |
| 46+ years | 103 | 2 (2 - 3) | 2 (1 - 3) | 2 (2 - 3) |
| ***Gender:*** | ***829*** |  |  |  |
| Female | 801 | 2 (2 - 3) | 2 (1 - 3) | 2 (2 - 3) |
| Male | 28 | 2 (1.75 - 3) | 2 (1 - 3) | 2 (2 - 3) |
| ***Highest level of VN education:*** | ***808*** |  |  |  |
| Diploma (or equivalent) | 588 | 2 (2 - 3) | 2 (1 - 3) | 2 (2 - 3) |
| Bachelor's/Postgraduate degree | 220 | 2 (2 - 3) | 2 (1 - 3) | 2 (2 - 3) |
| ***Highest level of non-VN education:*** | ***812*** |  |  |  |
| None | 333 | 2 (2 - 3) | 2 (1 - 3) | 2 (2 - 3) |
| NVQ/Diploma | 335 | 2 (2 - 3) | 2 (1 - 3) | 2 (2 - 3) |
| BSc | 127 | 2 (2 - 3) | 2 (1 - 3) | 2 (2 - 3) |
| MSc/PhD | 17 | 3 (2 - 3) | 2 (1.25 - 3) | 2 (2 - 3) |
| ***Time qualified (years):*** | ***835*** |  |  |  |
| <1 year | 33 | 2 (2 - 3) | 2 (1 - 3) | 2 (2 - 3) |
| 1-6 years | 345 | 2 (2 - 3) | 2 (1 - 3) | 2 (2 - 3) |
| 7-14 years | 218 | 2 (2 - 3) | 2 (1 - 3) | 2 (2 - 3) |
| 15+ years | 239 | 2 (2 - 3) | 2 (1 - 3) | 2 (2 - 3) |
| ***Employment status:*** | ***827*** |  |  |  |
| Full time | 636 | 2 (2 - 3) | 2 (1 - 3) | 2 (2 - 3) |
| Part time | 191 | 2 (2 - 3) | 2 (1 - 3) | 2 (2 - 3) |
| ***Role:*** | ***835*** |  |  |  |
| Student veterinary nurse | 64 | 3 (2 - 3) | 2 (1 - 3) | 2 (2 - 3) |
| Veterinary nurse | 479 | 2 (2 - 3) | 2 (1 - 3) | 2 (2 - 3) |
| Senior nurse | 220 | 2 (2 - 3) | 2 (1 - 3) | 2 (2 - 3) |
| Specialised nurse | 56 | 2 (2 - 3) | 2 (1 - 3) | 2 (2 - 3) |
| Senior manager | 16 | 2 (2 - 3) | 2 (1 - 2) | 2 (2 - 3) |
| ***Practice type:*** | ***824*** |  |  |  |
| GP/Charity | 526 | 2 (2 - 3) | 2 (1 - 3) | 2 (2 - 3) |
| Hospital | 141 | 2 (2 - 3) | 2 (1 - 3) | 2 (2 - 3) |
| Referral Hospital/Mixed General and Referral | 90 | 2 (2 - 3) | 2 (2 - 3) | 2 (2 - 3) |
| University Hospital | 39 | 3 (2 - 3) | 2 (2 - 3) | 2 (2 - 3) |
| Emergency Clinic | 39 | 2 (1 - 3) | 2 (1 - 3) | 2 (2 - 3) |
| ***Main species cared for:*** | ***829*** |  |  |  |
| Small Animal | 721 | 2 (2 - 3) | 2 (1 - 3) | 2 (2 - 3) |
| Equine | 20 | 3 (2 - 4) | 2 (1.75 - 3) | 2 (2 - 3) |
| Mixed | 88 | 2 (2 - 3) | 2 (1 - 3) | 2 (2 - 3) |

IQR = interquartile range
